# Relation between Negative Symptoms and Core Stability as an Indicator of Functional Exercise Capacity in Schizophrenia

**DOI:** 10.1192/j.eurpsy.2023.2232

**Published:** 2023-07-19

**Authors:** E. I. Hoşgelen, N. Bozbıyık, Ö. Akgül, F. Şimşek, N. Gelecek, K. Alptekin

**Affiliations:** 1 Neurosciences, Dokuz Eylül University; 2 Physiotherapy, Izmir Bakırçay University; 3 Department of Psychology, İzmir Democracy University; 4 Psychiatry, Bakırcay University Education and Research Hospital, Community Mental Haalth Service; 5 Musculoskeletal Physiotherapy Department, Dokuz Eylül University Physical Theraphy and Rehabilitation; 6 Department of Psychiatry, Dokuz Eylul University School of Medicine, İzmir, Türkiye

## Abstract

**Introduction:**

The core refers to the lumbo-pelvic complex located at the center of gravity of the body.Core stability has a crucial role in sudden balance change and body movements(Zemkova et al. Front. Physiol 2022; 13).The more stabilized core muscles indicate better movements and body balance.Therefore, core stability is related with exercise performance, falling risk and falling fear.However, patients with schizophrenia have lower motivation and capability to exercise compared to normal population that may result decrease in core stability.

**Objectives:**

The aim of this study is to determine the relation between core stability,functional exercise capacity and negative symptoms including especially anhedonia, motivation, psychosocial functioning in schizophrenia patients.As our knowledge,this is the first study to detect the core stability in schizophrenia.

**Methods:**

Participants of the study were recruited from the Community Mental Health Service of Çigli Education and Research Hospital. Twenty-six individuals diagnosed as schizophrenia according to DSM-V criteria were included into the study.Symptom severity was evaluated with Positive and Negative Syndrome Scale(PANSS), psychosocial functioning was assessed with Personal and Social Performance Scale(PSP),depression was assessed using Calgary Depression Scale for Schizophrenia(CDSS),avoidance motivation assessed with Behavioral Inhibition System and Behavioral Activation System (BIS/BAS),social and physical pleasure were measured via Revised Social Anhedonia Scale (RSAS) and Revised Physical Anhedonia Scale Functional (RPAS) exercise capacity was assessed by 6-minute walking test(6MWT) and core stability was assessed using Mcgill Core Endurance Tests(MCET).Patients who exercise regularly, having metabolic diseases or comorbid psychiatric disorder were excluded from the study.The data was analyzed by IBM SPSS 24 with Pearson correlation test.

**Results:**

MCET scores were found to be moderately correlated to PSP scores (r=.45,p=.025) and BIS sensitivity was moderately correlated to psychosocial functioning (r=-.42,p=.035).Six-MWT scores were negatively correlated with BIS (r=-.51, p=.019),CSDC (r=-.47, p=.035) and PANSS negative subscale (r=-.42, p=.042).

**Conclusions:**

In this study core stability was found to be related to psychosocial functioning.Also,patients having negative symptoms and depression showed lower functional exercise capacity.Lower scores in social functioning and higher behavioral inhibition sensitivity may be related to psychosocial dysfunctioning and negative symptoms of schizophrenia.However, appropriate core stability training and physical exercise may help individuals with schizophrenia to improve these skills.This may lead patients to exercise,have better social performance,self-care and lower avoidance behavior.

**Disclosure of Interest:**

E. I. Hoşgelen: None Declared, N. Bozbıyık: None Declared, Ö. Akgül: None Declared, F. Şimşek: None Declared, N. Gelecek: None Declared, K. Alptekin Grant / Research support from: TUBITAK, Consultant of: Abdi Ibrahim, Abdi İbrahim Otsuka, Janssen, Ali Raif

